# Role of the Advanced Practice Provider in Clinical Trials: Contributions to the Management of Patients Receiving Inotuzumab Ozogamicin

**Published:** 2017-09-01

**Authors:** Mary Alma Welch, Joanne C. Ryan, Ilene Ann Galinsky

**Affiliations:** 1 MD Anderson Cancer Center, Houston, Texas;; 2 Pfizer Inc, New York, New York;; 3 Dana Farber Cancer Institute, Boston, Massachusetts

Successfully conducting oncology clinical trials requires the involvement of various research personnel. Advanced practice providers (APPs) are integral members of the team and play a vital role in identifying and enrolling appropriate patients, educating participants, and providing supportive care throughout the process. 

Clinical research is crucial for developing new, more effective treatments and helping us to learn more about the efficacy and safety of existing therapies and novel combinations. Planning and conducting a clinical study involve several steps (Figure), and the various phases of clinical studies are designed to assess the appropriate dose, efficacy, and safety of new therapies (Table 1). In addition to advancing medical treatments and ensuring patient safety, maintaining data integrity is an important goal of clinical studies. 

**Figure:**
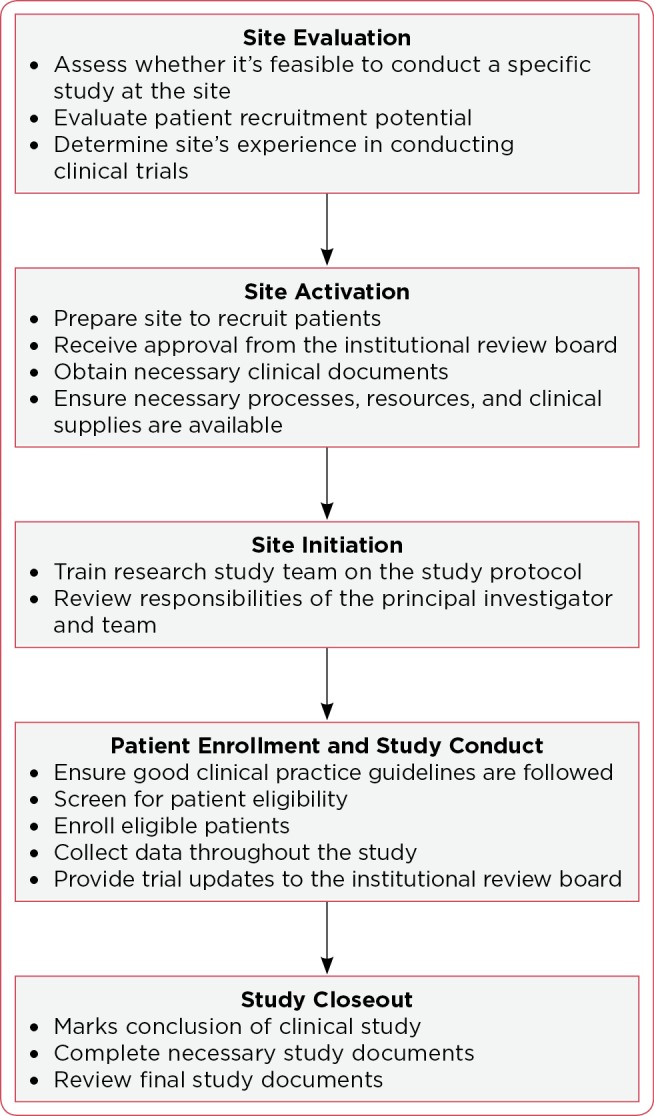
Planning and conducting a clinical trial.

**Table 1:**
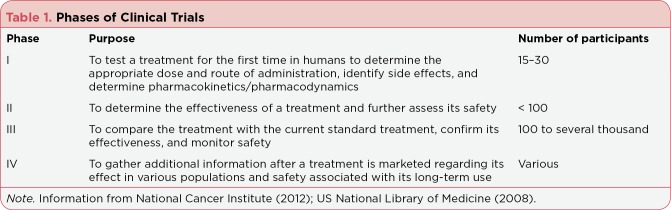
Phases of Clinical Trials

In this article, we highlight the role of the APP in clinical research through the experiences of a physician assistant and a nurse practitioner, both of whom have been active in the management of patients with relapsed or refractory (R/R) acute lymphoblastic leukemia (ALL) treated with inotuzumab ozogamicin (InO) vs. the investigator’s choice of chemotherapy in the phase III INO-VATE trial.

## UNDERSTANDING ACUTE LYMPHOBLASTIC LEUKEMIA AND INOTUZUMAB OZOGAMICIN

Acute lymphoblastic leukemia is a rare, life-threatening disease affecting lymphoid progenitor cells that occurs in adults and children (Curran & Stock, 2015; Pui, Robison, & Look, 2008). Typically, adults have worse outcomes than pediatric patients (Curran & Stock, 2015), with complete remission (CR) rates roughly between 60% and 90% with current induction therapies (Bassan & Hoelzer, 2011; DeAngelo et al., 2015). However, some patients are refractory to initial treatment (Thomas et al., 2004), and as many as 60% of adult patients relapse in the first 5 years after diagnosis (Kantarjian et al., 2004).

Standard treatment options for adult patients with R/R ALL are limited, and patient outcomes are poor (Oriol et al., 2010). The only potentially curative treatment following relapse is an allogeneic stem cell transplant. However, only a fraction of patients qualifies for this treatment, because in addition to finding an appropriate donor (Tavernier et al., 2007), eligible patients must have achieved CR (Gökbuget et al., 2012; Thomas et al., 1999). Therefore, there is an unmet need for additional clinical research and treatment options for adults with R/R ALL.

The role of APPs in caring for patients with ALL is particularly important given the patients’ need for supportive care, frequent transfusion of blood products, and management of side effects. In our institutions, the APPs often see patients more frequently than the physicians.

In a large referral center with a dedicated leukemia department, education about disease biology and clinical course is a mainstay of the orientation process for APPs. Additional treatment-specific instruction occurs in several ways: interaction and collaboration with the principal investigator and research nurse, attendance at site-initiation visits and in-services provided by the study sponsors, attendance at national meetings and continuing education seminars, and day-to-day hands-on management of patients. A team approach is essential to appropriately monitor and report responses and side effects, so future patients benefit from the most up-to-date information. 

The number of cancer therapies that target molecular factors necessary for cancer growth and progression is rapidly increasing (Ciavarella, Milano, Dammacco, & Silvestris, 2010). Among them is inotuzumab ozogamicin, an antibody-drug conjugate comprising a humanized anti-CD22 monoclonal antibody conjugated to the cytotoxic antibiotic calicheamicin, which is currently being studied in patients with ALL (Shor, Gerber, & Sapra, 2015). Once bound by InO, CD22 (a cell-surface glycoprotein expressed on the surface of B cells of most patients [> 90%] with B-cell ALL; Boué & LeBien, 1988) is internalized into lysosomes, and calicheamicin is released to bind to the minor groove of DNA and induce double-strand cleavage with resultant apoptosis (Bouchard, Viskov, & Garcia-Echeverria, 2014; Shor et al., 2015). A phase II study in patients with R/R ALL showed InO was well tolerated and active, supporting further research in a phase III study (Kantarjian et al., 2013). 

## ROLE OF ADVANCED PRACTICE PROVIDERS IN THE INO-VATE TRIAL

Advanced practice providers participated in all aspects of the phase III INO-VATE trial, an open-label, two-arm study (ClinicalTrials.gov identifier NCT01564784) evaluating the clinical activity and safety of InO compared with standard intensive chemotherapy (cytarabine, fludarabine, and granulocyte colony-stimulating factor, cytarabine plus mitoxantrone, or high-dose cytarabine; Kantarjian et al., 2016). Advanced practice providers functioned as subinvestigators and were registered with the US Food and Drug Administration, the sponsoring pharmaceutical company, and their institutions’ research offices.

Once potential patients were identified in the clinic or hospital, the APPs worked closely with the attending physician and research team to recruit them and review their eligibility according to protocol guidelines. Typically, this process involved ensuring a patient fulfilled all of the clinical requirements of participation, such as adequate organ function, performance status, and ability to comply with study-required testing. Once eligibility was determined, the physician was responsible for obtaining consent, whereas the APPs played a major role in facilitating the consent process by educating the patients and answering patients’ questions about how InO works, what monitoring tests are required, and what potential side effects to look for and how they can be managed. In this role, APPs were on the front line in ensuring the well-being and safety of study participants. 

In both of our institutions, patients are seen more frequently by APPs than by physicians for the routine review of labs and physical assessments and management of side effects. After a patient began treatment on the INO-VATE trial, APPs assumed a major role in direct patient care. Patients with ALL who receive therapy require frequent laboratory work and monitoring, sometimes as often as three to four times a week.

Common hematologic adverse events (AEs) observed with InO include thrombocytopenia, neutropenia, anemia, febrile neutropenia, and leukopenia; common nonhematologic AEs include nausea, pyrexia, diarrhea, and headache (Kantarjian et al., 2016). Advanced practice providers also monitored patients for important liver-related AEs, including increased aspartate aminotransferase and alanine aminotransferase levels, hyperbilirubinemia, and veno-occlusive disease. Advanced practice providers assessed AEs, including reviewing laboratory results and radiographic studies; determined the grading and causality of any AEs; and ordered concomitant medications or transfusions for appropriate management. Advanced practice providers also informed physicians of any AE patients experienced to aid with decisions regarding treatment delays, dose reductions, and treatment discontinuations. 

In addition, APPs performed procedures such as bone marrow aspirates/biopsies, Ommaya taps, and lumbar punctures as clinically indicated for monitoring disease status and response to therapy. They also performed respiratory examinations, neurologic examinations, and complete physical examinations; administered intrathecal therapies; as well as prepared qualified patients for transplant. Among the most frequent interventions provided were intravenous fluid and electrolyte replacement; management of prophylactic antimicrobials; transfusion of packed red blood cells and platelets; and treatment of common complaints, such as nausea, vomiting, diarrhea, anorexia, and fatigue. Advanced practice providers also assessed the patient’s need for growth factors and assessed when to coordinate central nervous system prophylaxis. The responsibilities of APPs in providing patient care and monitoring not only apply to clinical trials, but also to the general care of any patient with a hematologic malignancy.

During clinical trials, APPs interact with, collaborate with, and educate other health-care professionals and clinical trial team members (Table 2). During the INO-VATE trial, APPs functioned as a resource for other clinical trial team members and for nurses caring for the patients. The APPs also served as liaisons between patients and the clinical study coordinator/research nurse to ensure assessments and other protocol-specific activities were conducted as required. Moreover, APPs played a vital role in facilitating a smooth transition to stem cell transplant in those patients who were eligible. They worked with the attending physician and pharmacist to determine the appropriate timing for the last dose of InO and coordinated the patient handoff from one team to another. Throughout the trial, it was important for APPs to communicate with the principal investigator and study coordinator/research nurse to ensure patients were managed per the study protocol and all necessary data were documented appropriately. Moreover, it was important for all clinical trial team members, including APPs, to work together as a team to ensure any changes to the study protocol were communicated to the appropriate parties.

**Table 2:**
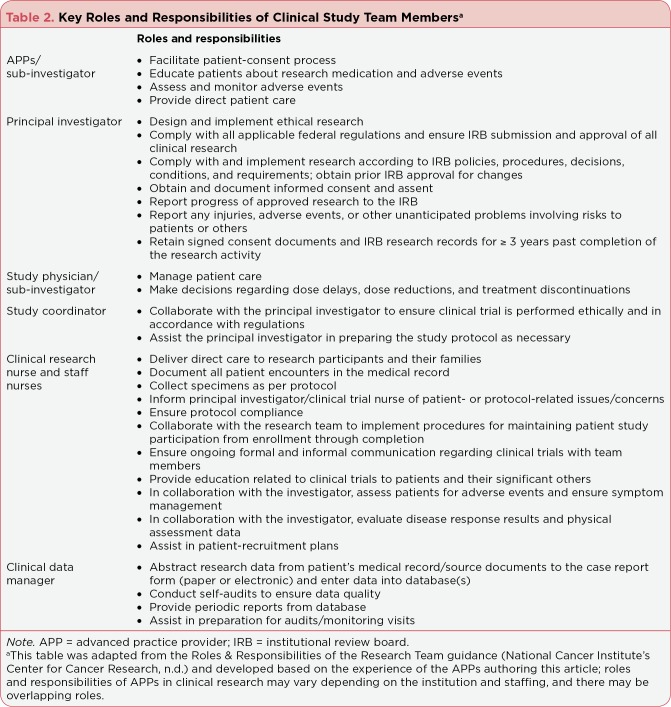
Key Roles and Responsibilities of Clinical Study Team Members^a^

The role of APPs in the INO-VATE trial serves as an example of the valuable part they play throughout the clinical research process. They are integral members of the clinical research team, working to ensure patient safety and the successful conduct of the trial.

## ROLE OF ADVANCED PRACTICE PROVIDERS AFTER THE INO-VATE TRIAL

The responsibilities of APPs are not limited to patient care throughout the clinical research process but continue after the end of a clinical trial.

In the initially published efficacy results of the INO-VATE trial, which included 218 patients (Kantarjian et al., 2016), InO was associated with a significantly higher response rate than standard chemotherapy, both in remission and minimal residual disease negativity (Table 3). Minimal residual disease is defined as < 0.01% marrow blasts (Kantarjian et al., 2016) and is considered a surrogate marker for outcomes and survival (Knechtli et al., 1998; Lonial & Anderson, 2014). More patients in the InO arm proceeded to stem cell transplant than those who received standard chemotherapy (41% vs. 11%).

**Table 3:**
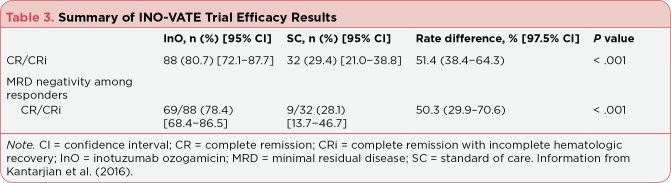
Summary of INO-VATE Trial Efficacy Results

A total of 259 patients were included in the safety population. For patients receiving InO vs. standard chemotherapy, the incidence of grade ≥ 3 thrombocytopenia was lower (37% vs. 59%), there were fewer platelet transfusions (64% vs. 95%), and the incidence of liver toxicity and veno-occlusive disease was higher (11% vs. 1%; Kantarjian et al., 2016). 

Understanding the efficacy and safety findings of the INO-VATE trial allows APPs to better educate and treat patients and to inform other health-care professionals about this promising therapeutic option. The experiences APPs gained from the INO-VATE trial also are invaluable for treating patients receiving InO through the compassionate-use program or investigator-initiated studies. Moreover, the knowledge APPs gained from the INO-VATE trial is useful beyond caring for patients administered InO and is also applicable to treating patients receiving other antibody-drug conjugate therapies.

## CONCLUSIONS

Through their integral involvement in clinical trials, APPs contribute to advancements in therapeutic options. During the INO-VATE trial, APPs played a pivotal role by helping patients navigate consent forms; providing safe and innovative patient care, including managing infusion reactions, liver toxicity, myelosuppression, and other side effects associated with InO; and collaborating with other clinical trial team members. Through this experience, APPs learned about the importance of antibody-drug conjugate therapy, gained a wealth of experience, and positioned themselves to play an important role in educating their peers about the use of InO in the treatment of patients with ALL. 

Inotuzumab ozogamicin was approved by the FDA on August 17, 2017, for the treatment of adults with relapsed or refractory B-cell precursor ALL. The APPs who worked in the InO trial will now be able to bring their valuable experience to the nonclinical trial patients.

**Acknowledgments**

The INO-VATE trial reviewed in this article is a clinical study sponsored by Pfizer Inc. Editorial support was provided by Anny Wu, PharmD, of Complete Healthcare Communications, LLC, and was funded by Pfizer Inc.
